# Supplementary material for: Versatile nanobody-based approach to image, track and reconstitute functional Neurexin-1 in vivo
Source: Nat Commun. 2024 Jul 18;15:6068. doi: 10.1038/s41467-024-50462-2 (PMC11258300; doi:10.1038/s41467-024-50462-2)
Supplement: Supplementary file 1 — Supplementary Information file [file 41467_2024_50462_MOESM1_ESM.pdf]

Supplementary Information:

Versatile nanobody-based approach to image, track and reconstitute functional Neurexin-1 *in vivo*

Authors:

Rosario Vicidomini[1#], Saumitra Dey Choudhury[1,2#], Tae Hee Han[1], Tho Huu Nguyen[1], Peter Nguyen[1], Felipe Opazo[3,4], and Mihaela Serpe[1]*

Affiliations

[1] Section on Cellular Communication, *Eunice Kennedy Shiver* National Institute of Child Health and Human Development, NIH, Bethesda, MD, 20892, USA.

[2] Present address: Centralized Core Research Facility-Microscopy, All India Institute of Medical Sciences, New Delhi 110029, Delhi, India

[3] Department of Neuro and Sensory Physiology, University Medical Center Göttingen, 37075 Göttingen, Germany

[4] NanoTag Biotechnologies GmbH, Rudolf-Wissell-Str. 28A 37079, Göttingen, Germany

[#]These authors contributed equally to this work

***Correspondent Author**:

Mihaela Serpe

Eunice Kennedy Shriver National Institute of Child Health and Human Development

National Institutes of Health

35 Convent Drive, Bldg. 35, Room 1C-1016

Bethesda, MD, USA, 20892

E-mail: mihaela.serpe@nih.gov

Phone: 301-443-3795

Supplementary Fig. 1

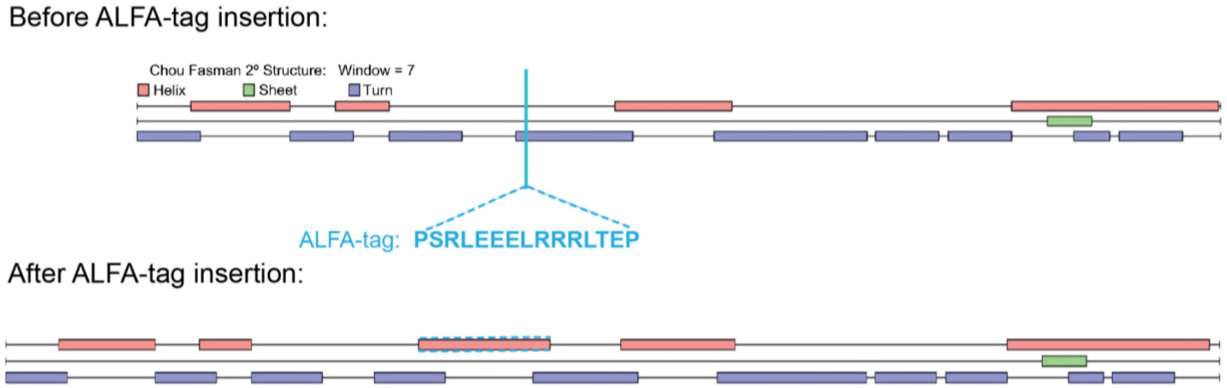

Supplementary Fig. 1. Secondary structure prediction for the intracellular domain of *Drosophila melanogaster* Nrx-1 before and after the ALFA tag insertion.

The protein analysis toolbox of the MacVector sequence analysis application (version 18.5) was used for the analysis of predicted secondary structure. The results obtained with the Chou Fasman algorithm are shown, as indicated.

Supplementary Fig. 2

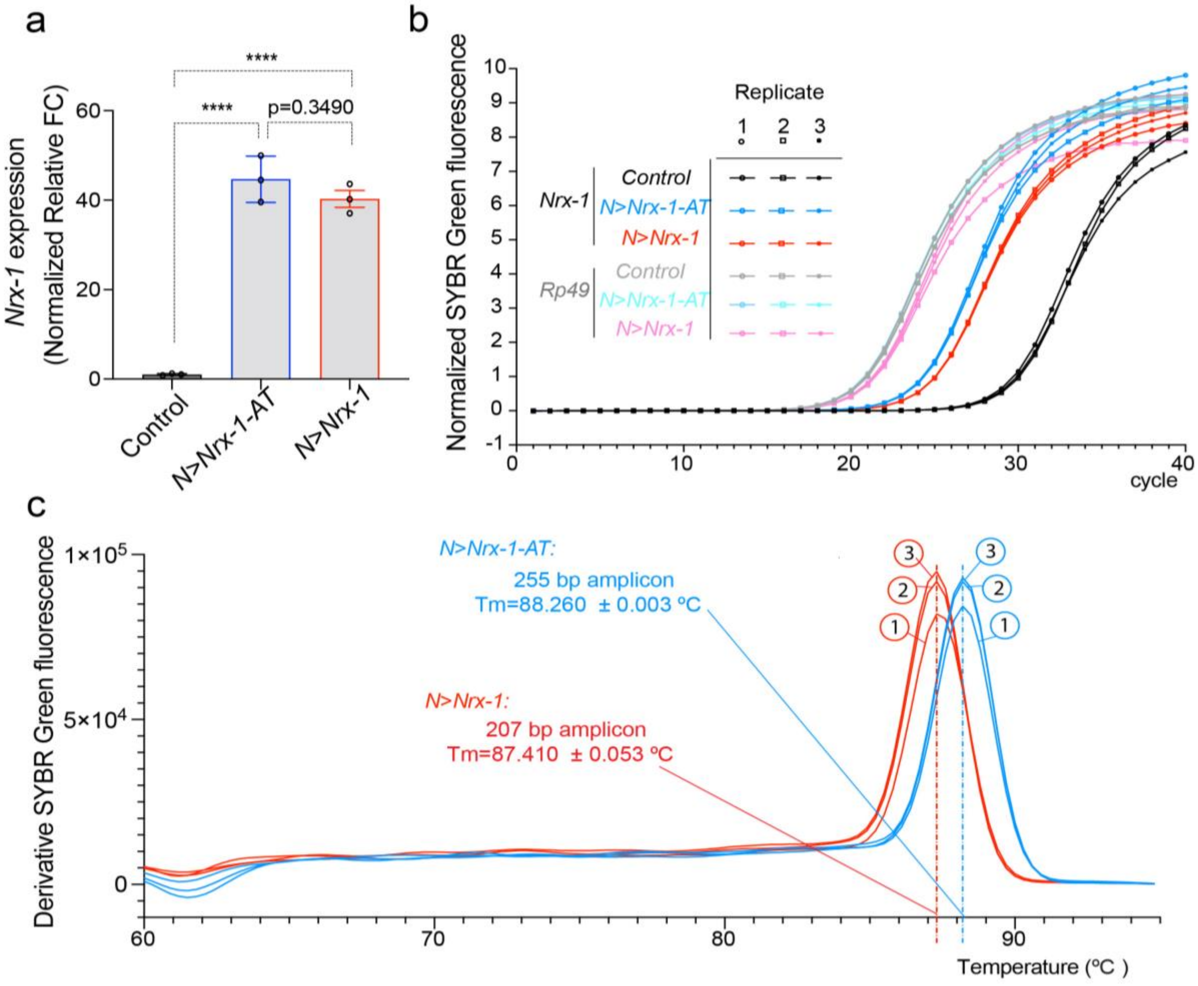

Supplementary Fig. 2. qPCR analysis of *Nrx-1* mRNA expression.

(**a**) Fold changes (FC) in *Nrx-1* mRNA expression levels, normalized to *RP49* mRNA, are shown for third instar larvae of different genotypes as indicated.

(b) Amplification curves: Normalized SYBR Green fluorescence as a function of PCR cycle numbers. *Rp49* mRNA was used as an internal control. Results from three different experiments are shown.

(**c)** The melting curves, measured at the end of the 40th amplification cycle, capture the difference between the two amplicons, (i) 207 bp for *Nrx-1* mRNA and (ii) 255 bp for *Nrx-1-AT* mRNA.

Data are represented as mean ±SEM. **** p<0.0001; ns, p=0.3490.

Genotypes: control (*BG380-Gal4/+;*), *N>Nrx-1 (tag/no tag)* (*BG380-Gal4/+; UAS-Nrx-1 tag/no tag/+).*

Supplementary Fig. 3

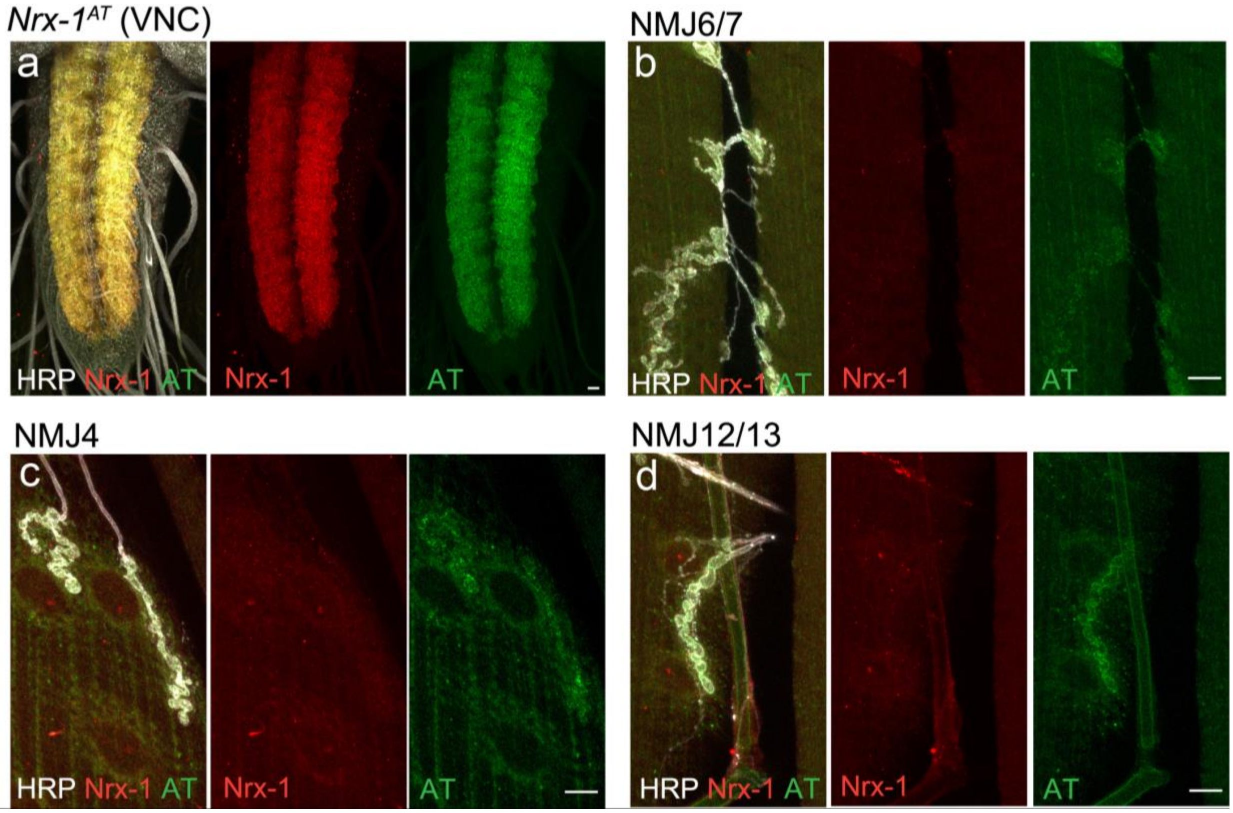

Supplementary Fig. 3. Detection of endogenously edited Nrx-1^{AT}.

(**a**-**d**) Confocal images of VNCs (**a**) or various NMJs (**b**-**d**) of *Nrx-1^{AT}* third instar larvae labeled for Nrx-1 (red), ALFA tag (green) and HRP (white). Conventional anti-Nrx-1 antibodies detect endogenous levels of synaptic Nrx-1^{AT} within the VNC; however, these antibodies could not capture the low abundant Nrx-1^{AT} at NMJ terminals, even after amplification with secondary antibodies. In contrast, the fluorescently labeled FluoTag-X2 anti-ALFA detected synaptic Nrx-1^{AT} in a single immunohistochemistry step in both VNC and multiple NMJs.

Scale bars: 10 µm.

Supplementary Fig. 4

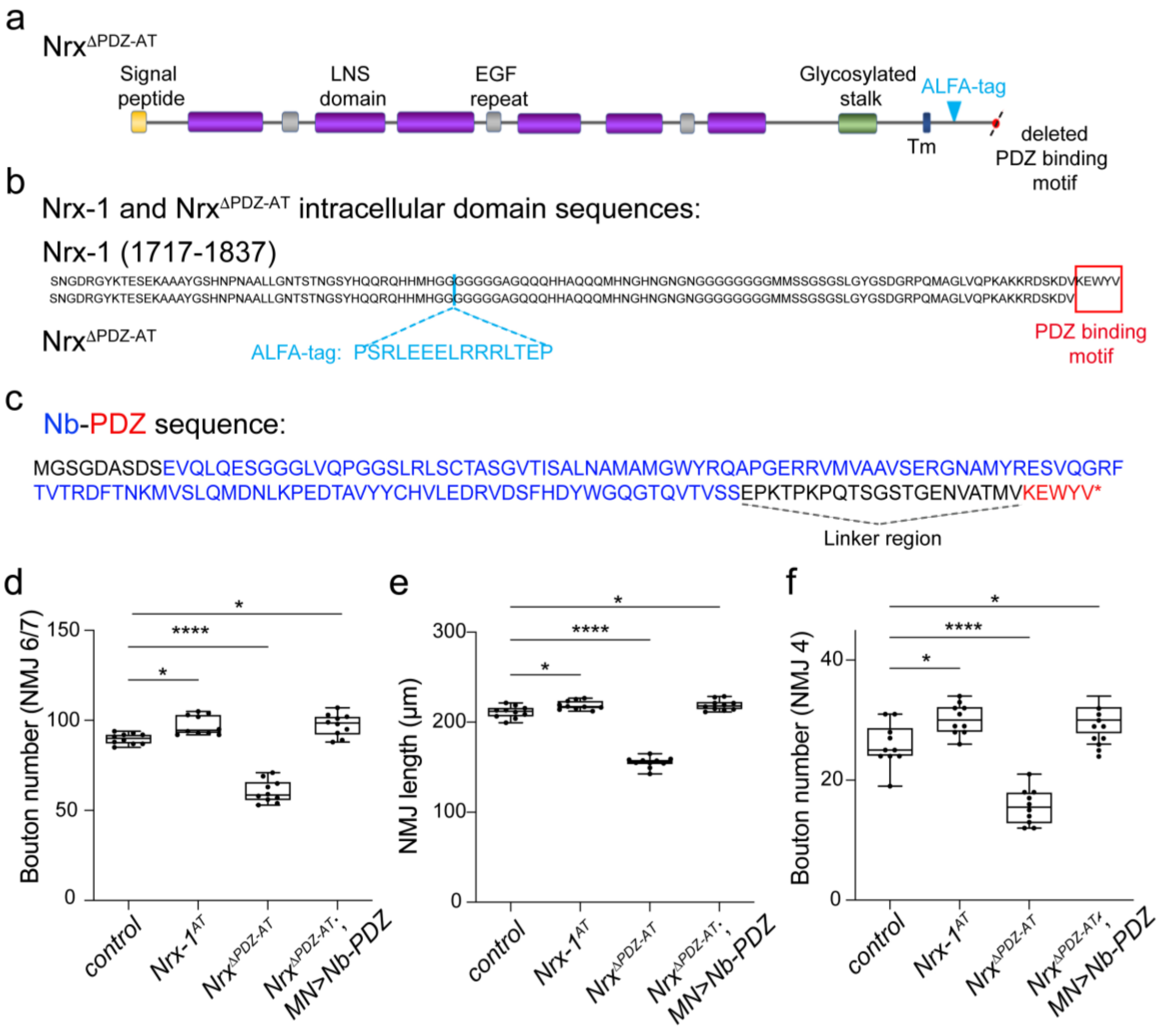

Supplementary Fig. 4. Insertion of ALFA tag at endogenous *Nrx-1* locus does not impair the NMJ growth.

(**a**) Diagram illustrating the Nrx$^{\Delta PDZ\text{-}AT}$ domain organization. The AT insertion site and the deletion of the PDZ binding motif are indicated.

(**b**) The intracellular domain sequences for endogenous Nrx-1 and modified Nrx$^{\Delta PDZ\text{-}AT}$.

(**c**) Sequence of the Nb-ALFA-PDZ chimera. Note the overhang residues (shown in black) in between the Nb-ALFA (blue) and PDZ binding motif (red) sequences.

(**d-f**) Quantification of bouton number (**d** and **f**) and NMJ length (**e**) in the indicated genotypes. Data are represented as mean ±SEM (one-way ANOVA with Tukey's multiple comparisons); **** $p<0.0001$, * $p<0.05$. The boxes expand from first to third quartile, and the whiskers from minimum to maximum values; the center lines mark the mean values. Source data are provided as a Source Data file.

Supplementary Fig. 5

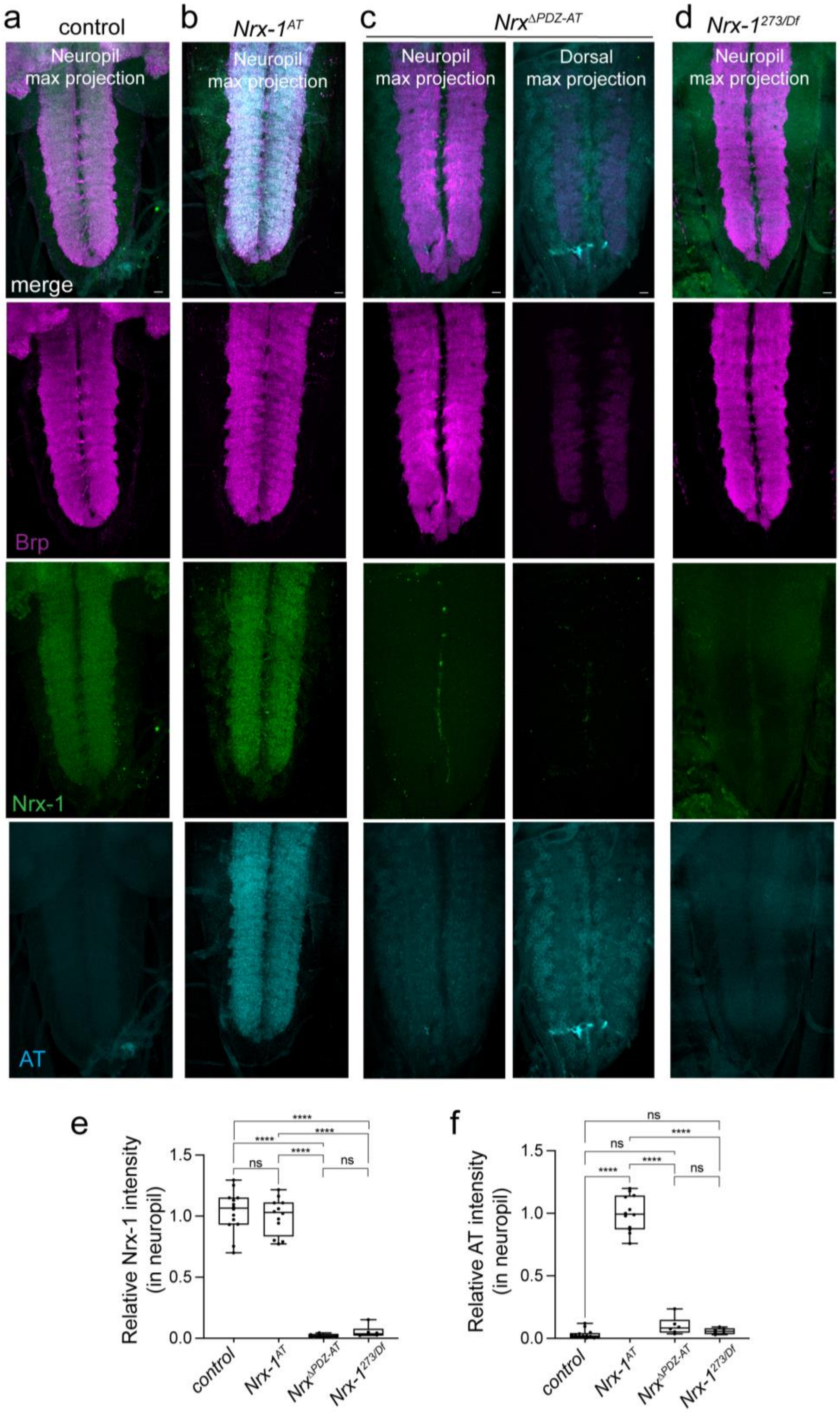

Supplementary Fig. 5. Quantification of Nrx-1 levels in the ventral nerve cord

(**a-d**) Confocal images of third instar larval VNC of various genotypes stained for Nrx-1 (green), Brp (magenta), and AT (cyan). Maximum intensity projections covering the neuropil or dorsal regions as indicated, are shown at the top; different individual channels are showed below. These experiments were repeated three times with similar results. Scale bars: 10 µm.

(**e-f**) Quantitative analysis of Nrx-1 (e) or AT (f) fluorescence intensities normalized to Brp signal. No significant change of Nrx-1 signal intensity was observed between control (w^{1118}) and the *Nrx-1*^{AT} edited line.

Data are represented as mean ±SEM (one-way ANOVA with Tukey's multiple comparisons); **** $p<0.0001$, ns, $p>0.05$. The boxes expand from first to third quartile, and the whiskers from minimum to maximum values; the center lines mark the mean values. Source data are provided as a Source Data file.

Supplementary Fig. 6

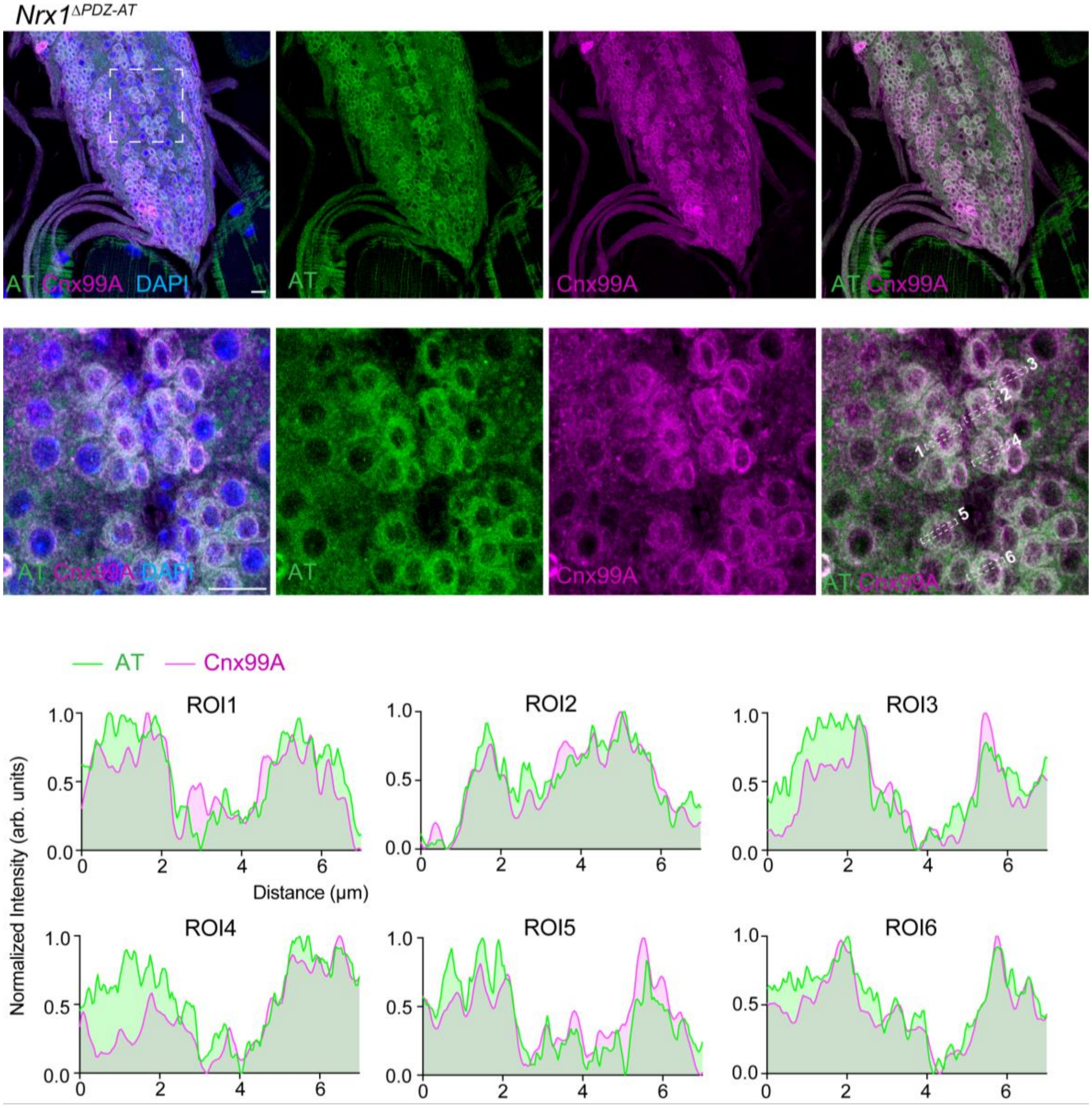

Supplementary Fig. 6. Retention of Nrx$^{\Delta PDZ\text{-}AT}$ in ER/ early Golgi compartments.

Maximum intensity projections of confocal images of third instar larvae *Nrx$^{\Delta PDZ\text{-}AT}$* VNCs labeled for AT (green), Calnexin-99A (magenta) and DAPI (blue).

To estimate the co-localization between AT and Calnexin-99A signals, we selected six linear regions of interest (ROIs) spanning different motor neuron cell bodies as indicated and plotted the normalized fluorescence intensity profiles along the ROIs length. The similarity between AT and Calnexin-99A profiles indicate that Nrx$^{\Delta PDZ\text{-}AT}$ remains confined to the ER/early Golgi compartments.

Scale bars: 10 µm.

Supplementary Fig. 7

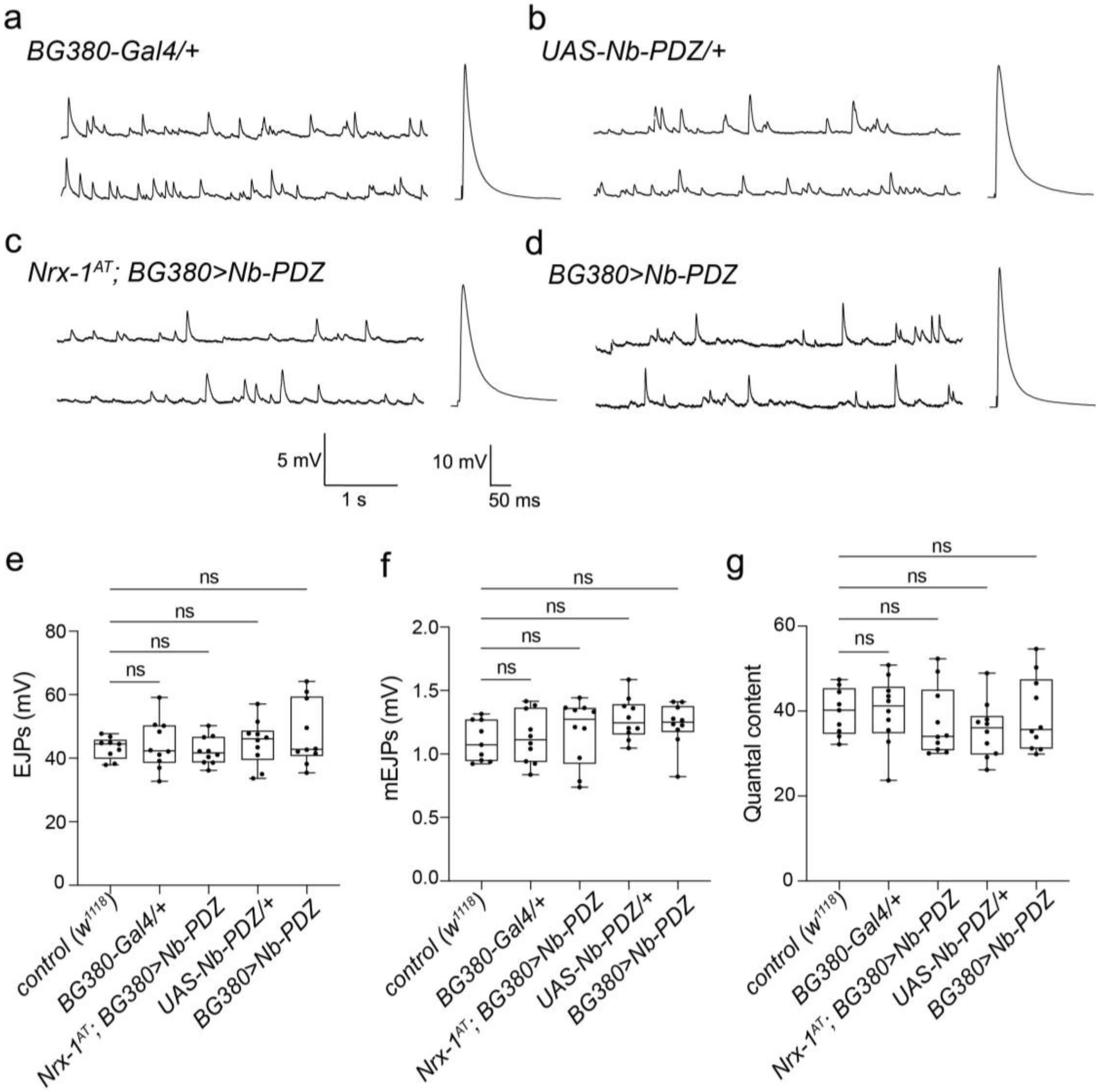

Supplementary Fig. 7. Overexpression of Nb-ALFA is not detrimental to the NMJ function. (**a-d**) Representative traces of miniature junctional potentials (mEJPs) and evoked junctional potentials (EJPs) in third instar larvae of indicated genotypes. The driver alone or the neuronal overexpression of Nb-PDZ in control backgrounds do not impact the electrophysiological properties recorded at these NMJs.

(**e-g**) Quantification of EJPs amplitude, mEJPs amplitude and Quantal Content of the indicated genotypes; n=9 for control and 10 for all other genotypes.

Data are represented as mean ±SEM (one-way ANOVA with Tukey's multiple comparisons); ns, p>0.05. The boxes expand from first to third quartile, and the whiskers from minimum to maximum values; the center lines mark the mean values. Source data are provided as a Source Data file.

Supplementary Fig. 8

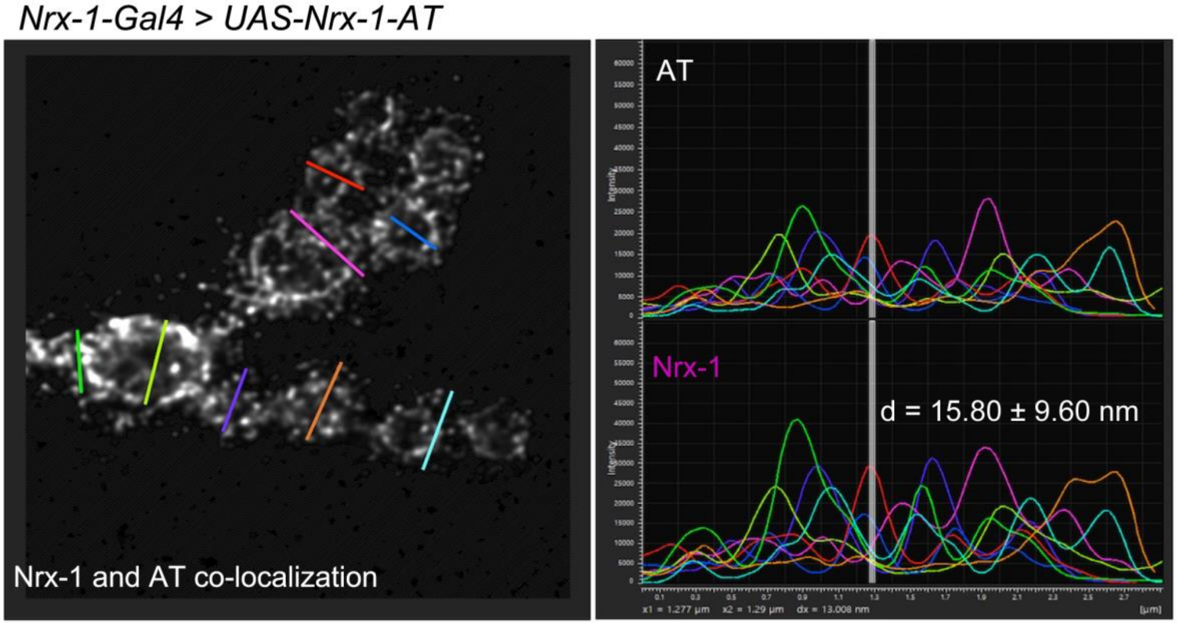

Supplementary Fig. 8. ALFA tag and Nrx-1 signals show good co-localization.

Plot profiles of eight randomly selected linear regions of interest (ROIs) reveal an average distance between Nrx-1 and ALFA tag positive peaks of 15.80 ± 9.60 nm, near the limit of STED microscopy. The results are presented as mean ±SEM.

The distance observed could be due to conventional amplification of the Nrx-1 signal vs. nanobody detection for the ALFA tag. Alternatively, this may reflect the limited ability to measure distances below 20 nm with STED. Nonetheless, the ALFA tag/nanobody system can be used for the precise localization of proteins of interest.

Supplementary Fig. 9

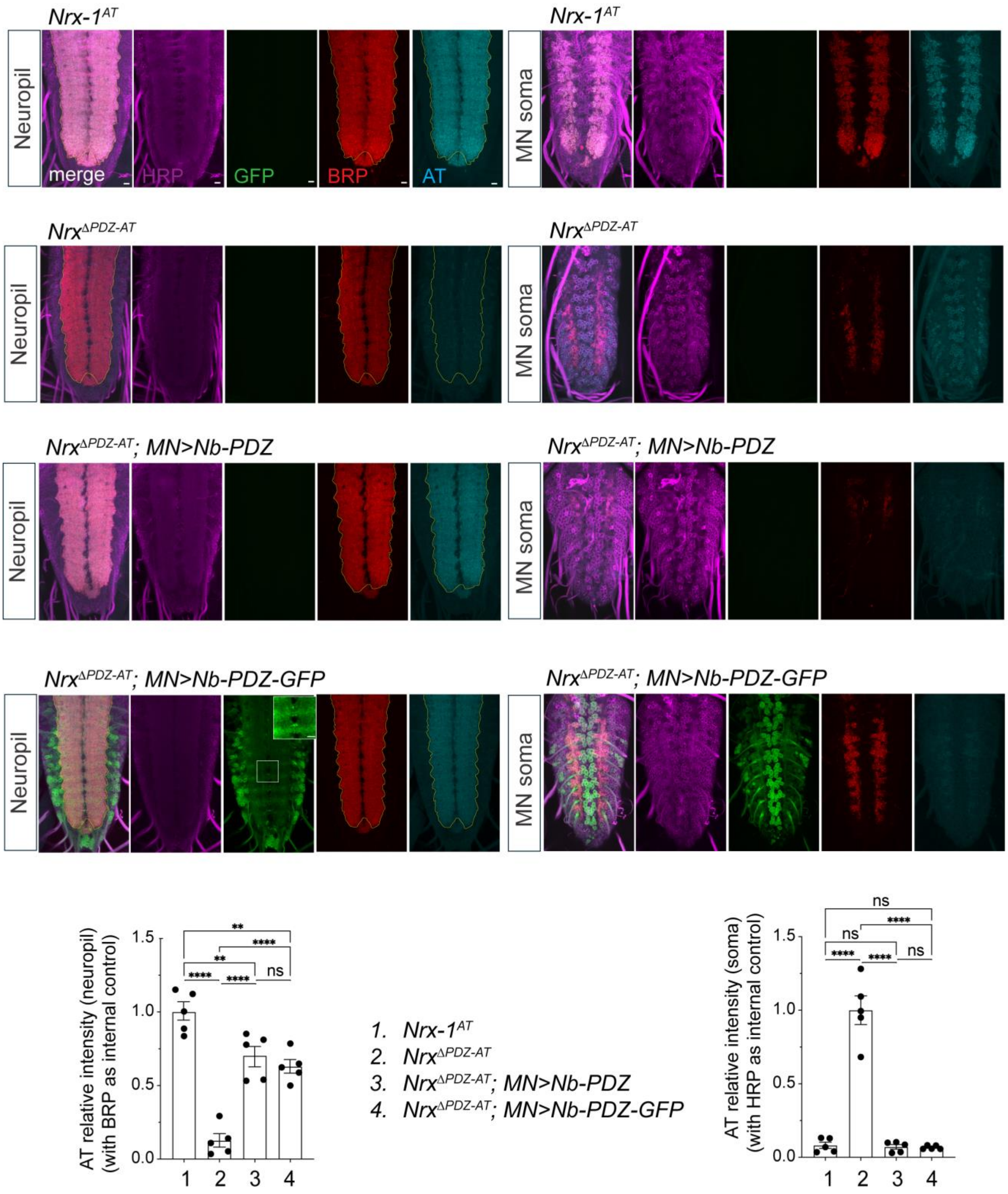

Supplementary Fig. 9. Nrx-1-AT redistributes the mScarlet-labeled intrabodies from cytosol to the cell membrane.

(**a-d**) Confocal images of third instar larval VNC of various genotypes stained for Hrp (magenta), Brp (red), GFP (green) and AT (cyan). The neuropil regions (left) and motor neuron soma (right) are marked. The detail in the GFP channel (d) shows strong GFP signal intensity in the neuropil; the GFP signal appears dim because of stronger lateral staining. Scale bars: 10 µm.

(**e-f**) Quantification of the relative AT fluorescence intensities in the neuropil (**e**) or in the motor neuron soma (**f**) normalized to the Brp intensities. The AT signal accumulates in the motor neuron soma only in the $Nrx^{\Delta PDZ\text{-}AT}$ larvae.

Data are represented as mean ±SEM (one-way ANOVA with Tukey's multiple comparisons); **** p<0.0001, ** p<0.01, ns, p>0.05. Source data are provided as a Source Data file.

Supplementary Fig. 10

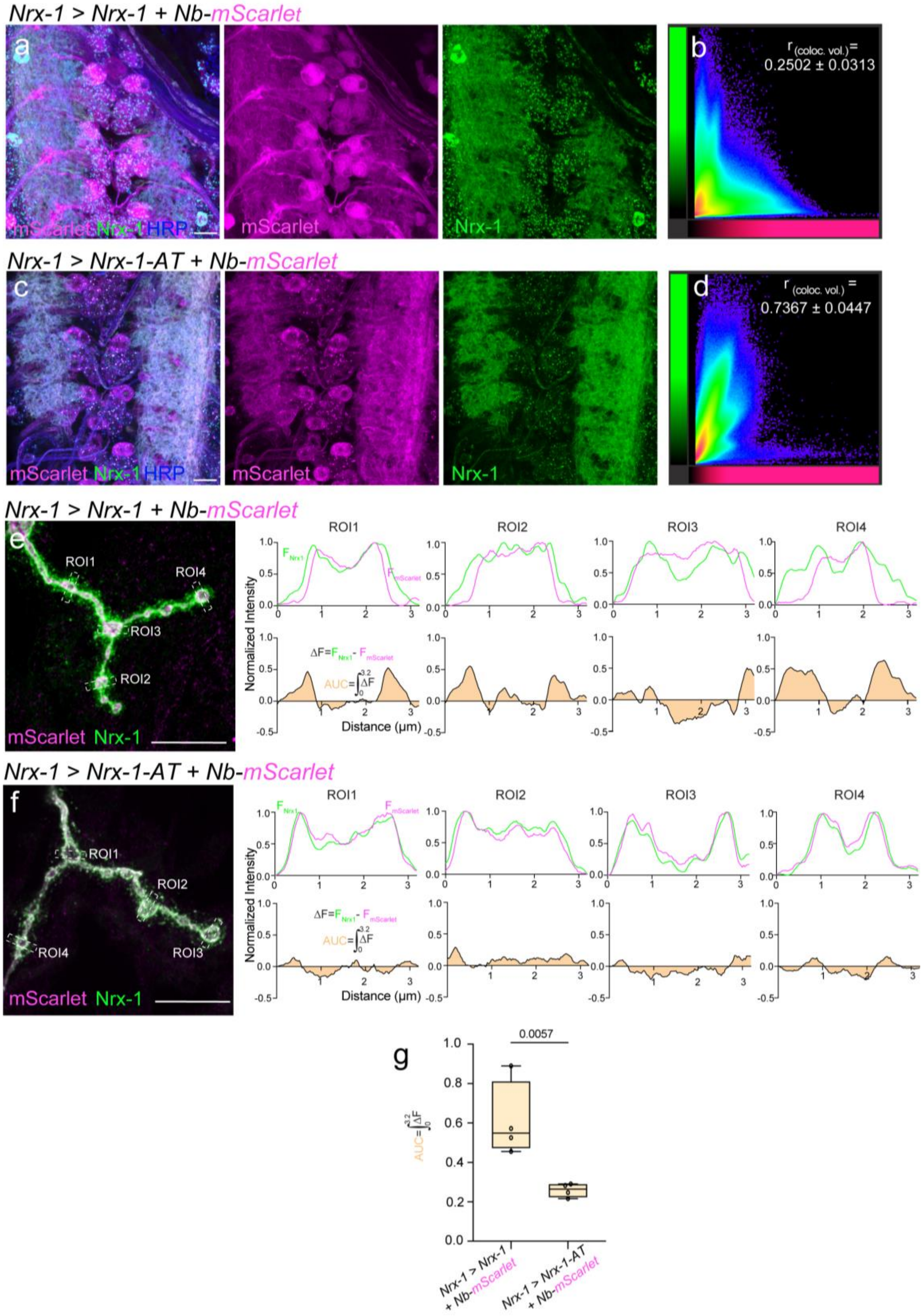

Supplementary Fig. 10. Nrx-1-AT redistributes the mScarlet-labeled intrabodies from cytosol to the cell membrane.

Maximum intensity projections of confocal images of VNCs (**a, c**) and synaptic boutons (**e, f**) from third instar larvae expressing cytosolic Nb-mScarlet together with either untagged Nrx-1 or Nrx-1-AT as indicated. All transgenes were expressed under the control of *Nrx-1-Gal4* and the specimens fixed and labeled for Nrx-1 (green), mScarlet (red) and HRP (blue).

(**b, d**) To quantify the co-localization between Nrx-1 and mScarlet channels in the VNCs, Pearson correlation coefficient was calculated within an HRP-selected mask. In the absence of an ALFA tag, Nb-mScarlet does not co-localize with Nrx-1 and instead localizes to the neuron soma ($r = 0.2502 \pm 0.0313$). In larvae expressing Nrx-1-AT, Nb-mScarlet mirrors the Nrx-1 distribution ($r = 0.7367 \pm 0.0447$).

To estimate the co-localization between Nrx-1 and mScarlet signals in synaptic boutons, we selected four linear regions of interest (ROIs) centered on synaptic boutons as indicated (**e, f**) and plotted the normalized fluorescence intensity profiles, F(Nrx1) and F(mScarlet), along the ROIs length. The difference, ΔF = F(Nrx1) - F(mScarlet), was calculated and the area under the curve (AUC) was determined. The AUC plots capture large differences between the Nrx1 and mScarlet profiles in the presence of the Nrx-1 and smaller differences in the presence of Nrx-1-AT ($p = 0.0057$) (**g**).

Scale bars: 10 µm.

Data are represented as mean ±SEM (unpaired t test). The boxes expand from first to third quartile, and the whiskers from minimum to maximum values; the center lines mark the mean values. Source data are provided as a Source Data file.

Supplementary Fig. 11

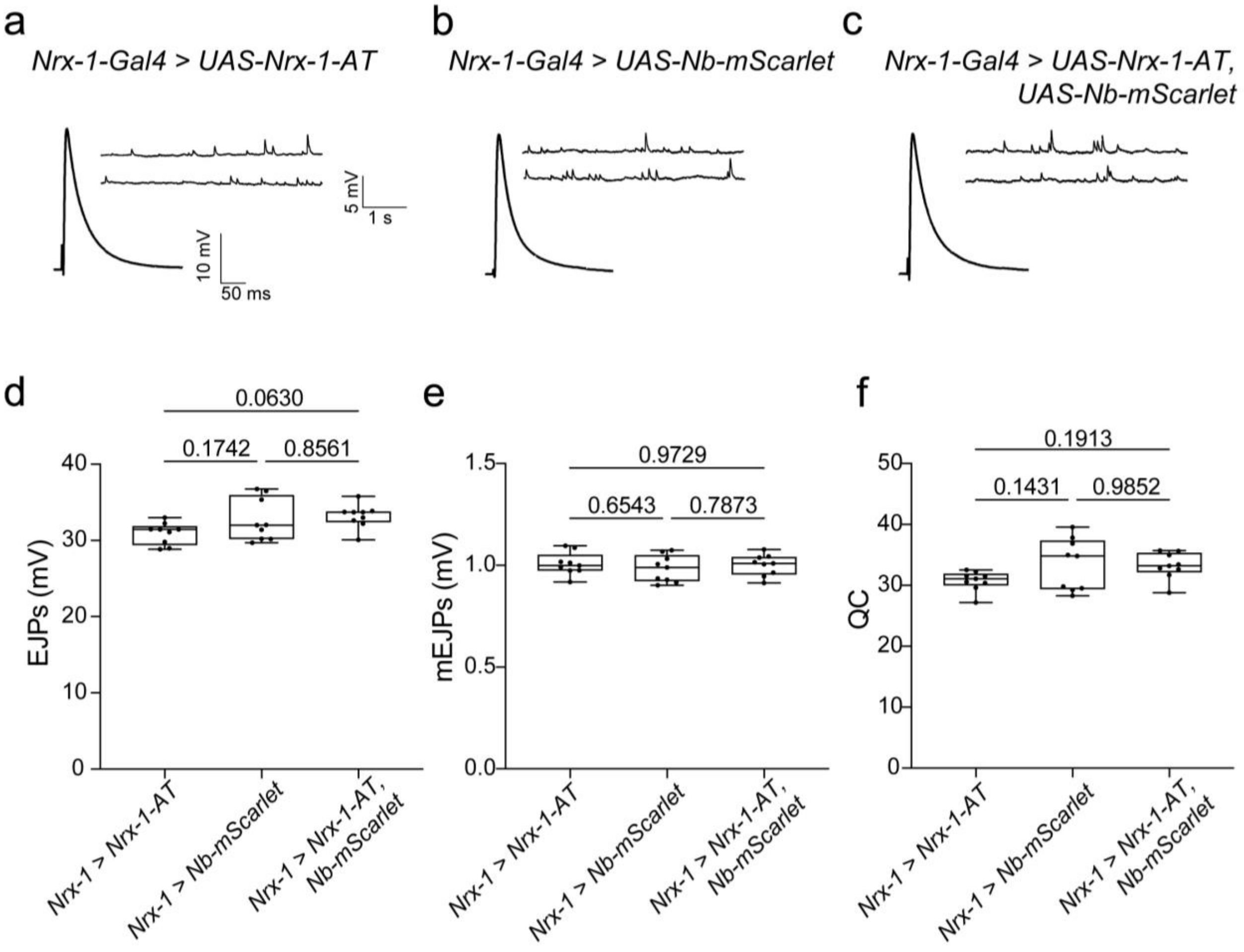

Supplementary Fig. 11. Overexpression of Nb-mScarlet does not disrupt NMJ function.

(**a-c**) Representative traces of miniature junctional potentials (mEJPs) and evoked junctional potentials (EJPs) in third instar larvae of indicated genotypes.

(**d-f**) Quantification of EJPs amplitude, mEJPs amplitude and Quantal Content (QC) of the indicated genotypes; n=9 for each genotype.

Data are represented as mean ±SEM (one-way ANOVA with Tukey's multiple comparisons). The boxes expand from first to third quartile, and the whiskers from minimum to maximum values; the center lines mark the mean values. Source data are provided as a Source Data file.

Supplementary Fig. 12

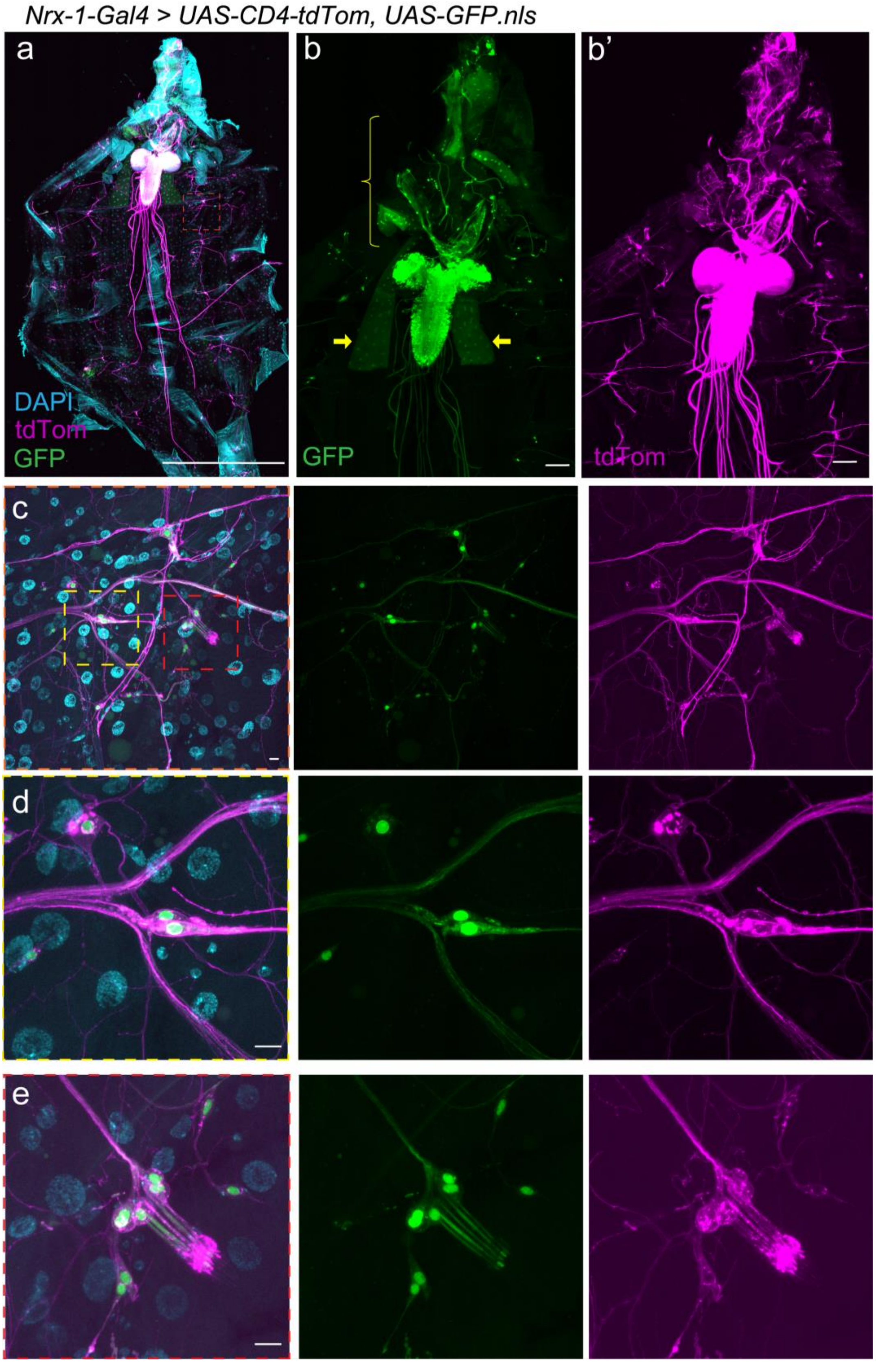

Supplementary Fig. 12. A *T2-Gal4* insertion at the *Nrx-1* locus reveals both pre- and postsynaptic Nrx-1 expression.

(**a-d**) Confocal micrograph of third instar larvae expressing *UAS-CD4-tdTom* and *UAS-GFP.nls* under the *Nrx-1-Gal4* control. Nrx-1 expression is not restricted to CNS neurons but is also present in some muscles, including thoracic muscles (**b**, accolade) and muscle 31, abdominal segment 1 (**b**, arrows). In addition, *Nrx-1-Gal4* induces expression of reporters in sensory neurons (**c**-**d**) and in the chordotonal organ (**e**).

Scale bars: 1000 µm (a), 100 µm (b-c) and 10 µm (d-e).

Supplementary Fig. 13

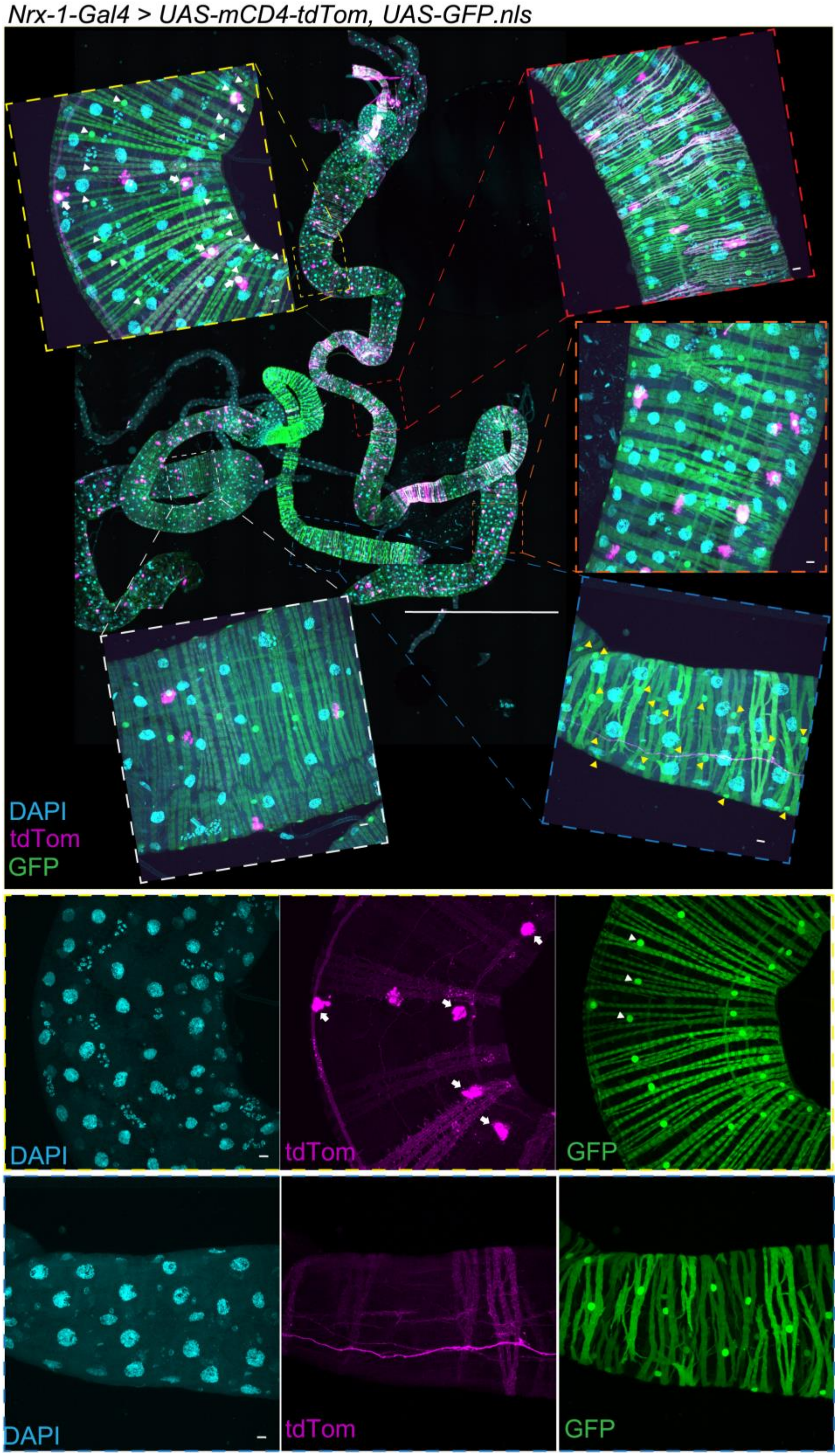

Supplementary Fig. 13. Nrx-1 is expressed in enteroendocrine cells and in visceral muscles ensheathing the entire intestine.

Confocal micrograph of a third instar larval gut expressing *UAS-CD4-tdTom* and *UAS-GFP.nls* under the *Nrx-1-Gal4* control.

Nrx-1-Gal4 drives expression of reporters in a subpopulation of enteroendocrine cells (marked by arrows), identified as sparse, non-polyploid cells in the gut, clearly separated from the islands of small stem cells and the large, polyploid enterocytes.

Nrx-1-Gal4 also drives expression in visceral muscles (arrowheads) along the entire intestine, from the proventriculus, to the midgut and to the hindgut.

Scale bars: 1000 µm and 10 µm (insets).

Supplementary Fig. 14

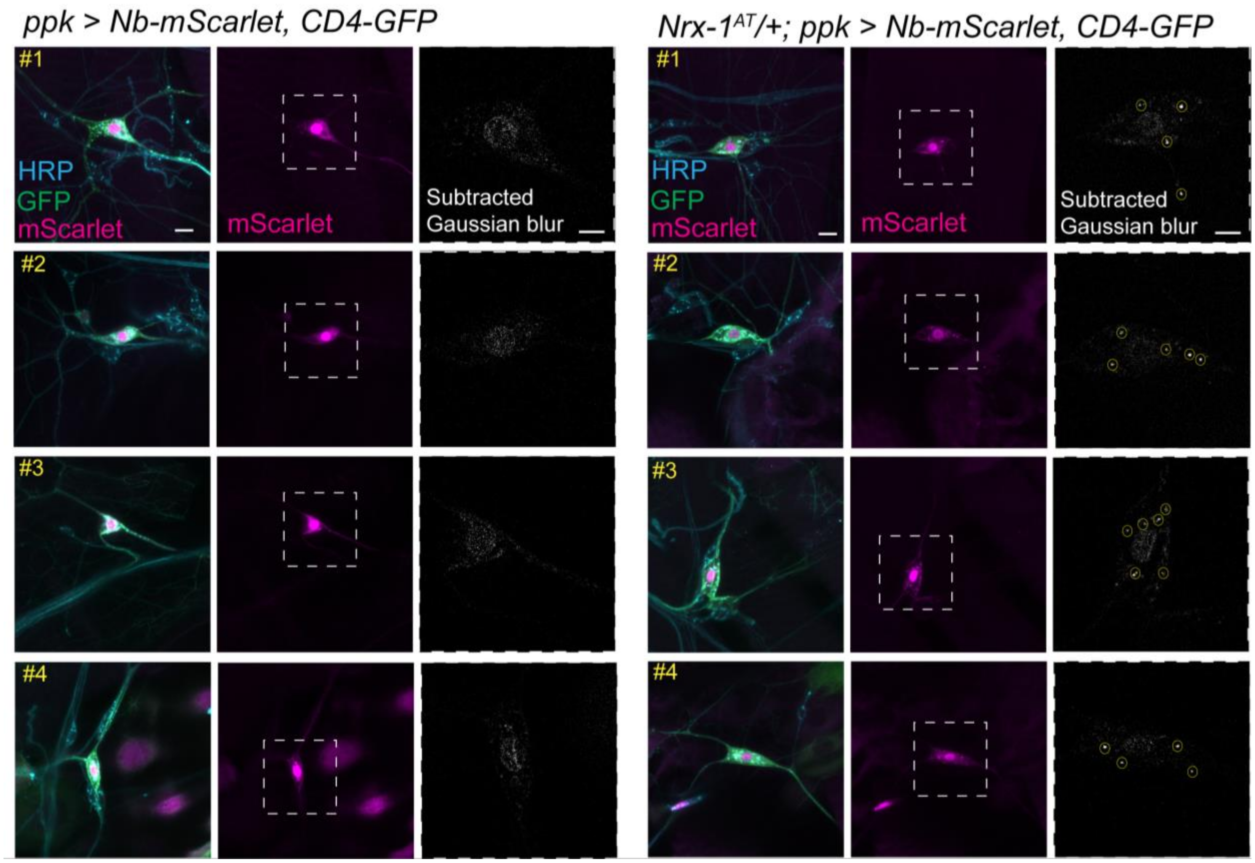

Supplementary Fig. 14. Compartment specific detection of endogenous Nrx-1 in subsets of sensory neurons.

Different examples of confocal images of larval sensory neurons from control (left column) or *Nrx-1-AT/+* heterozygous (right column) third instar larvae expressing cytosolic Nb-mScarlet and membrane-bound mCD4-GFP under the control of *ppk-Gal4*. The larval fillets were fixed and labeled for mScarlet (magenta), GFP (green) and HRP (cyan); the mScarlet signals were further filtered using Gaussian blur and image subtraction. All *Nrx-1-AT/+* animals show multiple mScarlet-positive vesicles, marked by yellow circles, but no such puncta could be detected in control animals.

Scale bars: 10 µm.

Supplementary Fig. 15

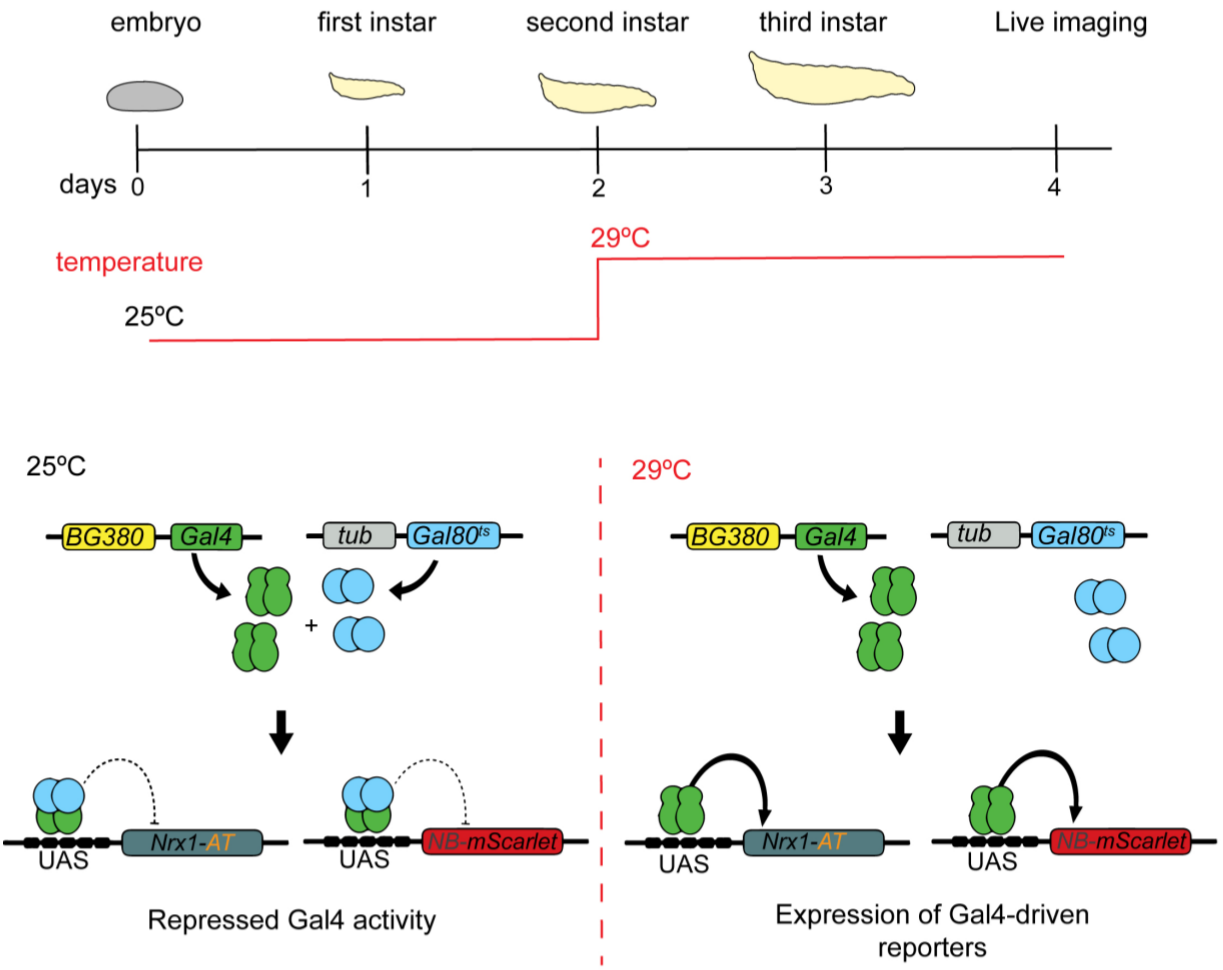

Supplementary Fig. 15. Tight control of Gal4-mediated expression using the Gal4/Gal80 system.

Diagram of the live imaging experimental setup. During development at permissive temperature (25°C), the Gal4 activity is repressed because of its binding to the Gal80 repressor. Once the developing animals are shifted to non-permissive temperatures (29°C), the Gal80[ts] can no longer bind GAL4 and its repressive function is lost. This temporally defined window of activity restricts the Gal4-induced transcription and refines the expression of target transgenes.

Supplementary Fig. 16

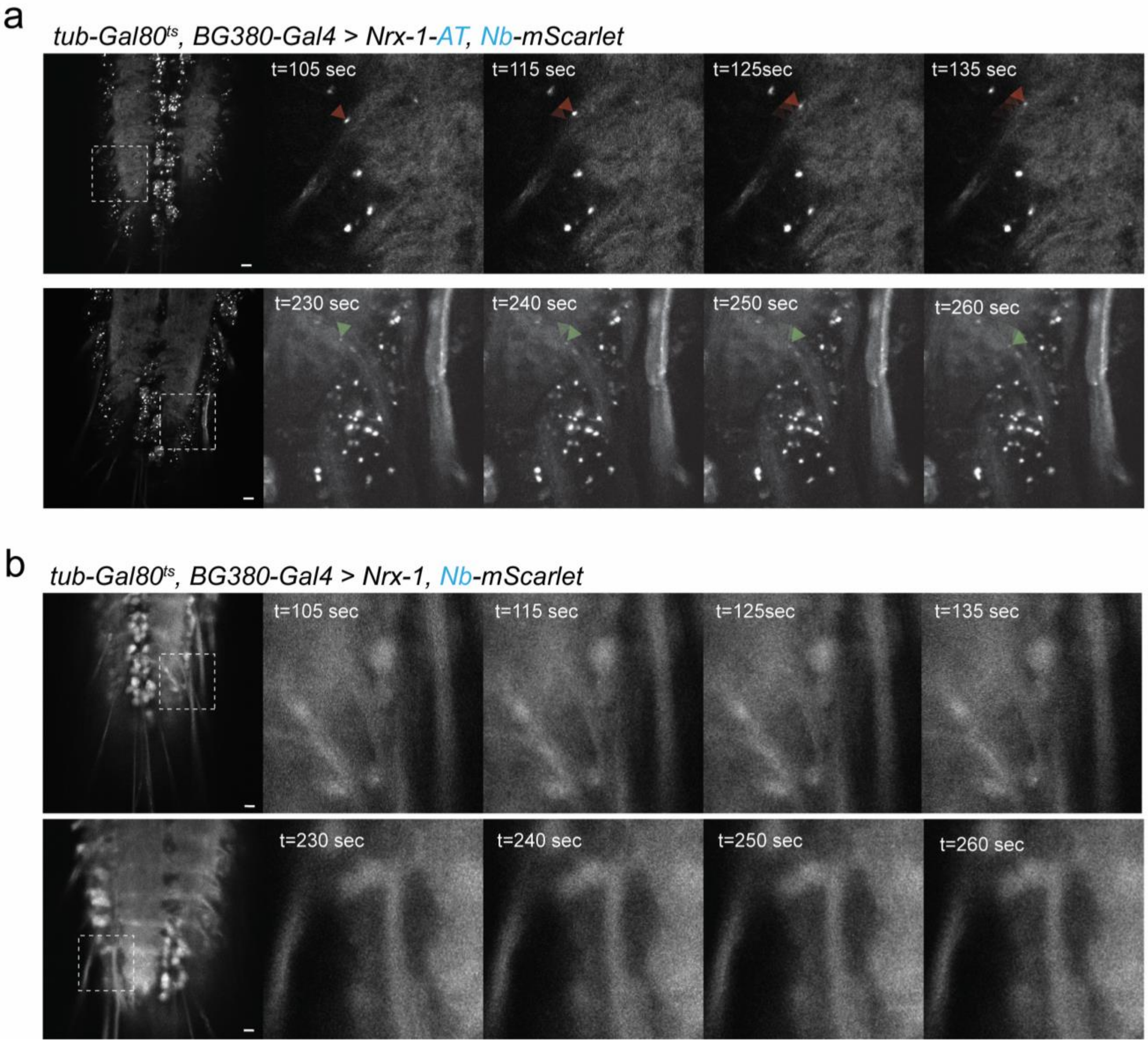

Supplementary Fig. 16. Live imaging of Nrx-1-AT trafficking.

(**a-b**) Representative confocal micrographs from time-lapse imaging from proximal axonal regions. Motor neurons received a short pulse of expression of *Nb-mScarlet* together with either *Nrx-1-AT* (**a**) or *Nrx-1* (**b**). The images are collected from the axonal bundles exiting the neuropil. The particles observed reside close to the neuron soma. Arrowheads indicate vesicles moving away (green) or towards (red) the VNC.

Scale bars: 10 µm.

Supplementary Fig. 17

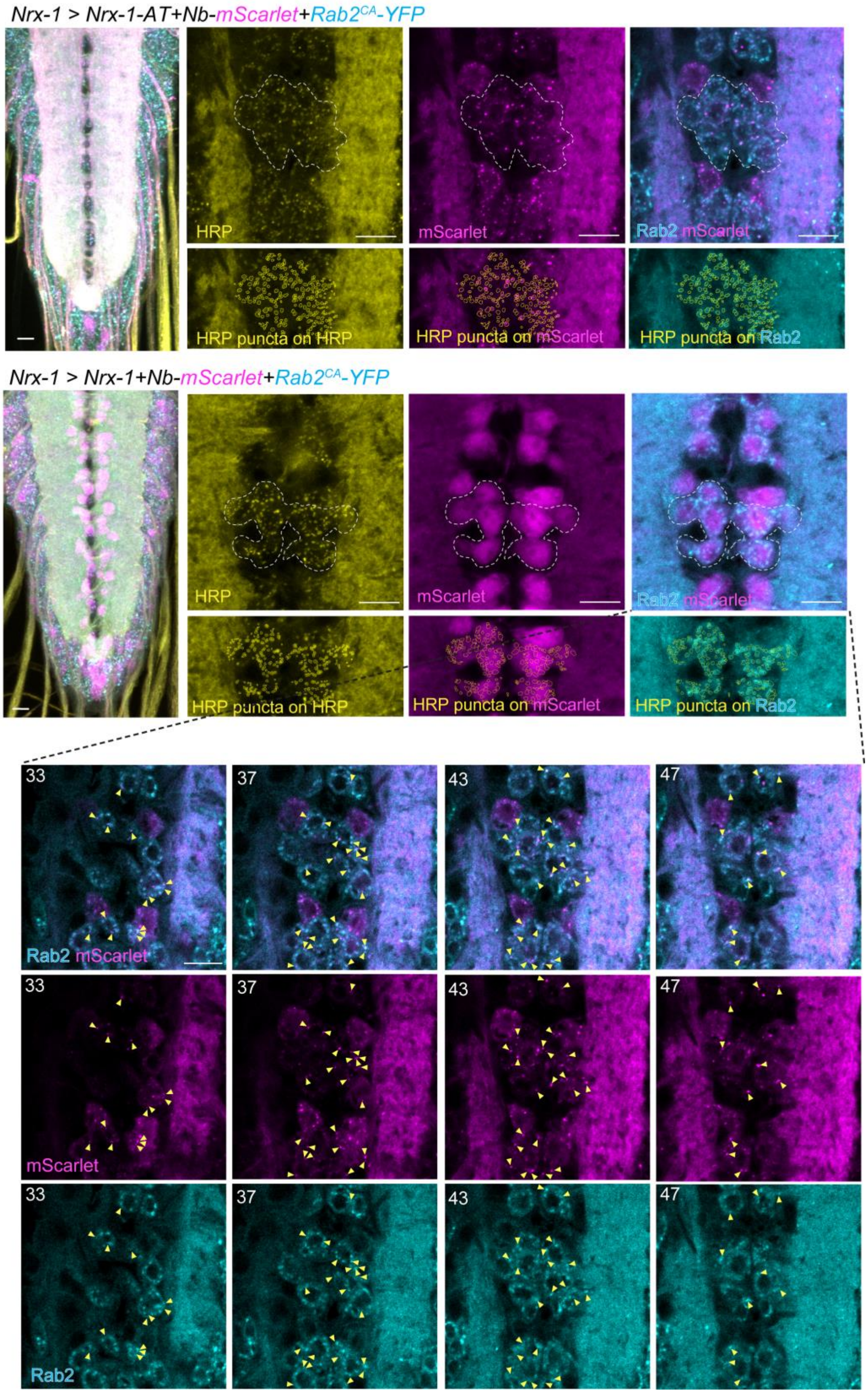

Supplementary Fig. 17 (extended Fig. 6). Most of the vesicles containing Nrx-1 (labeled via mScarlet) are Rab2-positive.

Confocal images of VNC from third instar larvae expressing *Nrx-1-AT* (upper part), or *Nrx-1* (lower part) together with *Nb-mScarlet* and *Rab2*^{CA}*-YFP* under the control of *Nrx-1-Gal4*. The specimens were fixed and stained together for mScarlet (magenta), YFP (cyan) and HRP (yellow) and imaged with the same confocal settings. Maximum projections of the full z-stack are shown in merged VNC images on the right. Of note, the mScarlet staining is much reduced in the neuropil in the negative control (lower VNC) and appears redistributed to the motor neuron soma. Maximum projections of z-planes containing the motor neuron soma are shown in individual or merged channels as indicated and the regions of interest comprising several motor neurons are marked. Puncta identified by segmentation (using Weka classifier and Stardist- see Materials and Methods) superimposed on individual channels are shown in the second row of panels.

Single planes of the Rab2 mScarlet z-stack are shown in the lower panels. Arrowheads indicate puncta positive for both Rab2 and mScarlet.

Scale bars: 10 µm.

Supplementary Table 1: Average values for the electrophysiological recordings

	Genotype	[Ca^{2+}] (mM)	mEJP (mV)	EJP (mV)	QC	R_{in} (MΩ)	Vm_{rest} (mV)	n
Fig. 2	*w^{1118}*	0.6	1.101 ± 0.053	40.258 ± 1.389	37.462 ± 2.776	6.477 ± 0.406	61.796 ± 0.693	9
Figs. 2 and S7	*BG380-Gal4/+ (MN-Gal4)*	0.6	1.132 ± 0.065	44.287 ± 2.453	39.987 ± 2.526	9.191 ± 0.710	63.702 ± 0.406	10
Fig. 2	*Nrx-1-/-*	0.6	1.724 ± 0.144	25.753 ± 1.331	15.690 ± 1.400	6.663 ± 0.504	62.419 ± 0.833	9
Fig. 2	*Nrx1-/- MN> Nrx-1*	0.6	1.021 ± 0.097	36.608 ± 0.503	38.251 ± 3.292	9.862 ± 1.165	63.403 ± 1.218	9
Fig. 2	*Nrx1-/- MN> Nrx-1-AT*	0.6	1.021 ± 0.073	35.364 ± 1.542	35.878 ± 2.695	9.033 ± 0.654	64.813 ± 1.396	9
Fig. 2	*Nrx1-/- MN> Nrx-1-GFP*	0.6	1.379 ± 0.166	30.252 ± 1.693	24.181 ± 2.681	8.233 ± 0.960	64.479 ± 1.479	9
Figs. 3 and S7	*w^{1118}*	0.6	1.102 ± 0.053	43.210 ± 1.158	39.809 ± 1.845	7.226 ± 0.659	62.918 ± 0.706	9
Fig. 3 and 4	*$Nrx\text{-}1^{AT}$*	0.6	1.103 ± 0.064	43.950 ± 3.496	40.341 ± 2.698	9.452 ± 0.720	63.761 ± 1.053	10
Fig. 3 and 4	*$Nrx^{\Delta PDZ\text{-}AT}$*	0.6	1.451 ± 0.108	28.954 ± 1.835	21.787 ± 3.483	8.617 ± 0.767	65.087 ± 1.591	10
Fig. 3	*$Nrx^{\Delta PDZ\text{-}AT}$ MN> Nb-PDZ*	0.6	1.082 ± 0.063	43.525 ± 2.012	41.457 ± 3.092	7.101 ± 0.809	65.690 ± 0.707	10
S7	*UAS-Nb-PDZ/+*	0.6	1.277 ± 0.052	44.921 ± 2.240	35.55 ± 2.094	7.436 ± 0.686	63.940 ± 0.509	10
S7	*MN> Nb-PDZ*	0.6	1.232 ± 0.055	47.641 ± 3.220	39.148 ± 2.791	9.150 ± 0.649	65.650 ± 0.772	10
S7	*Nrx^{AT} MN> Nb-PDZ*	0.6	1.175 ± 0.081	42.326 ± 1.387	37.457 ± 2.569	9.270 ± 0.609	65.311 ± 0.591	10
Fig. 4	*$Nrx^{\Delta PDZ\text{-}AT}$ MN> Nb-PDZ*	0.6	1.001 ± 0.030	42.216 ± 2.012	42.571 ± 2.403	6.797 ± 0.404	65.838 ± 0.528	11

Fig. 4	$Nrx^{\Delta PDZ\text{-}AT}$ *MN> Nb-PDZ-GFP*	0.6	1.472 ± 0.121	26.174 ± 1.319	18.788 ± 1.706	7.533 ± 0.571	65.467 ± 0.882	10
S11	*Nrx-1>Nrx-1-AT*	0.6	1.008 ± 0.019	30.921 ± 0.476	30.740 ± 0.540	8.054 ± 0.329	64.416 ± 0.410	9
S11	*Nrx-1>Nb-mScarlet*	0.6	0.983 ± 0.022	32.680 ± 0.928	33.436 ± 1.420	7.211 ± 0.408	64.180 ± 0.548	9
S11	*Nrx-1>Nrx-1-AT+ Nb-mScarlet*	0.6	1.001 ± 0.017	33.186 ± 0.515	33.211 ± 0.722	9.451 ± 0.455	65.923 ± 0.634	9

Supplementary Table 2: Experimental conditions for immunohistochemistry

	Fixation	Washes	Primary antibodies	Secondary antibodies
Fig. 1	Bouin, 3 min RT	PBS + 0.5% Triton X-100	Rb-anti-Nrx-1, 1:500	anti-Rb-Alexa568,1:200
			anti-ALFA-647, 1:500	-
			Ck-anti-GFP, 1:1,000	anti-Ck-Alexa488, 1:200
			o/n 4°C	o/n 4°C
Fig. 2	4% PFA, 20 min RT	PBS + 0.5% Triton X-100	M-anti-Csp, 1:1,000	anti-M-Alexa488
			anti-HRP-647, 1:1,000	-
			o/n 4°C	RT 1h
Fig. 3	Bouin, 3 min RT	PBS+ 0.1% Triton X-100	Rb-anti-Nrx-1, 1:500	anti-Rb-Alexa568,1:200
			anti-ALFA647, 1:500	-
			anti-HRP-FITC, 1:1000	-
			o/n 4°C	RT 1h
Fig. 4 (b-c)	4% PFA, 20 min RT	PBS + 0.5% Triton X-100	M-anti-Brp, 1:200	Anti-M-Alexa647, 1:200
			FluoTag-X4-anti-GFP, 1:500	-
			FluoTag-Q-anti-RFP, 1:500	-
			o/n 4°C	RT 1h
Fig. 4 (d-g)	Bouin, 3 min RT	PBS + 0.5% Triton X-100	Rb-anti-Nrx-1, 1:500	anti-Rb-Alexa568,1:200
			anti-ALFA647, 1:500	-
			Ck-anti-GFP, 1:1,000	anti-Ck-Alexa488, 1:200
			o/n 4°C	o/n 4°C
Fig. 5 (a-f)	4% PFA, 20 min RT	PBS + 0.5% Triton X-100	Rb-anti-Nrx-1, 1:500	anti-Rb-Alexa488,1:200
			anti-HRP-647, 1:1,000	-
			o/n 4°C	o/n 4°C
Fig. 5 (g-j)	4% PFA, 20 min RT	PBS + 0.5% Triton X-100	FluoTag-X4-anti-GFP, 1:500	
			FluoTag-Q-anti-RFP, 1:500	
			anti-HRP-647, 1:1000	
			o/n 4°C	
Fig. 6	4% PFA,		FluoTag-X4-anti-GFP, 1:500	

	20 min RT	PBS + 0.5% Triton X-100	anti-HRP-647, 1:1000	
			o/n 4°C	
S3	Bouin, 3 min RT	PBS + 0.1% Triton X-100	Rb-anti-Nrx-1, 1:500	anti-Rb-Alexa568,1:200
			anti-ALFA647, 1:500	-
			anti-HRP-647, 1:1000	-
			o/n 4°C	o/n 4°C
S5	Bouin, 3 min RT	PBS + 0.1% Triton X-100	Rb-anti-Nrx-1, 1:500	anti-Rb-Alexa488,1:200
			anti-ALFA-647, 1:500	-
			M-anti-BRP, 1:200	anti-M-Alexa568, 1:200
			o/n 4°C	o/n 4°C
S6	4% PFA, 20 min RT	PBS + 0.1% Triton X-100	M-anti-Cnx99A, 1:10	anti-M-Alexa568,1:200
			anti-ALFA488, 1:500	-
			o/n 4°C	o/n 4°C
S8	Bouin, 3 min RT	PBS + 0.5% Triton X-100	Rb-anti-Nrx-1, 1:500	anti-Rb-Alexa568,1:200
			anti-ALFA647, 1:500	-
			Ck-anti-GFP, 1:1,000	anti-Ck-Alexa488, 1:200
			o/n 4°C	o/n 4°C
S9	Bouin, 3 min RT	PBS + 0.1% Triton X-100	M-anti-Brp, 1:200	anti-M-Alexa568, 1:200
			anti-HRP-405, 1:1000	-
			FluoTag-X4-anti-GFP, 1:500	-
			anti-ALFA647, 1:500	-
			o/n 4°C	o/n 4°C
S10	4% PFA, 20 min RT	PBS + 0.5% Triton X-100	Rb-anti-Nrx-1, 1:500	anti-Rb-Alexa488,1:200
			anti-HRP-647, 1:1000	-
			o/n 4°C	o/n 4°C
S12	4% PFA, 20 min RT	PBS + 0.5% Triton X-100	FluoTag-X4-anti-GFP, 1:500	
			FluoTag-Q-anti-RFP, 1:500	
			o/n 4°C	
S13	4% PFA, 20 min RT	PBS + 0.5% Triton X-100	FluoTag-X4-anti-GFP, 1:500	
			FluoTag-Q-anti-RFP, 1:500	
			anti-HRP-647, 1:1000	
			o/n 4°C	
S14	4% PFA, 20 min RT	PBS + 0.5% Triton X-100	FluoTag-X4-anti-GFP, 1:500	
			FluoTag-Q-anti-RFP, 1:500	
			anti-HRP-647, 1:1000	
			o/n 4°C	
S17	4% PFA, 20 min RT	PBS + 0.5% Triton X-100	FluoTag-X4-anti-GFP, 1:500	
			anti-HRP-647, 1:1000	
			o/n 4°C	

Code for the analysis of puncta composition (utilized for Figure 6 and S17)

```
# Load required libraries
library(ggplot2)
library(ggExtra)
library(RColorBrewer)
library(cowplot)
library(ggpubr)

# Data Selection
selected_rows <- Summary_Table[Summary_Table$Genotype == "nrx>Rab2+Scarlet+Nrx1-
AT", ]
selected_rows2 <- Summary_Table[Summary_Table$Genotype == "nrx>Rab2+Scarlet+Nrx1-",
]

# Scatter Plot - selected_rows
p1 <- selected_rows %>%
  ggplot(aes(x = `Rab2 Intensity`, y = `Scarlet Intensity`)) +
  geom_point(aes(alpha = 0.03), color = "#69b3a2", size = 2) +
  geom_smooth(method = "lm", se = FALSE, size = 0.5, color = "black") +
  stat_regline_equation() +
  scale_color_manual(values = cols) +
  theme_bw() +
  theme(panel.border = element_blank(), panel.grid.major = element_blank(),
        panel.grid.minor = element_blank(), axis.line = element_line(colour = "black")) +
  theme(legend.position = "bottom") +
  labs(x = "Rab2 Intensity", y = "Scarlet Intensity")

p1
ggMarginal(p1, color = "black", type = "density", alpha = 0.1, size = 2, fill = "#69b3a2")

# Scatter Plot - selected_rows2
p2 <- selected_rows2 %>%
  ggplot(aes(x = `Rab2 Intensity`, y = `Scarlet Intensity`)) +
  geom_point(aes(alpha = 0.03), color = "black", size = 1) +
  geom_smooth(method = "lm", se = FALSE, size = 0.5) +
  stat_regline_equation() +
  scale_color_manual(values = cols) +
  theme_bw() +
  theme(panel.border = element_blank(), panel.grid.major = element_blank(),
        panel.grid.minor = element_blank(), axis.line = element_line(colour = "black")) +
  theme(legend.position = "bottom") +
  labs(x = "Rab2 Intensity", y = "Scarlet Intensity")

p2
ggMarginal(p2, color = "black", type = "density", alpha = 0.2, size = 2)

# Puncta Composition Analysis
num_HRPpuncta_Scarlet_zero <- sum(selected_rows$`Scarlet Intensity` == 0)
cat("Number of HRP puncta with zero Scarlet Intensity:", num_HRPpuncta_Scarlet_zero, "\n")
num_HRPpuncta_Scarlet_positive <- sum(selected_rows$`Scarlet Intensity` > 0)
```

```
cat("Number of HRP puncta with positive Scarlet Intensity:", num_HRPpuncta_Scarlet_positive,
"\n")
num_HRPpuncta_Rab2_zero <- sum(selected_rows$`Rab2 Intensity` == 0)
cat("Number of HRP puncta with zero Rab2 Intensity:", num_HRPpuncta_Rab2_zero, "\n")
num_HRPpuncta_Rab2_positive <- sum(selected_rows$`Rab2 Intensity` > 0)
cat("Number of HRP puncta with positive Rab2 Intensity:", num_HRPpuncta_Rab2_positive,
"\n")

num_Scarlet_Rab2_zero <- sum(selected_rows$`Scarlet Intensity` == 0 &
selected_rows$`Rab2 Intensity` == 0)
cat("Number of HRP puncta with zero Scarlet and Rab2 Intensity:", num_Scarlet_Rab2_zero,
"\n")
num_Scarlet_Rab2_positive <- sum(selected_rows$`Scarlet Intensity` > 0 &
selected_rows$`Rab2 Intensity` > 0)
cat("Number of HRP puncta with positive Scarlet and Rab2 Intensity:",
num_Scarlet_Rab2_positive, "\n")
num_Scarlet_zero_Rab2_positive <- sum(selected_rows$`Scarlet Intensity` == 0 &
selected_rows$`Rab2 Intensity` > 0)
cat("Number of HRP puncta with zero Scarlet and positive Rab2 Intensity:",
num_Scarlet_zero_Rab2_positive, "\n")
num_Scarlet_positive_Rab2_zero <- sum(selected_rows$`Scarlet Intensity` > 0 &
selected_rows$`Rab2 Intensity` == 0)
cat("Number of HRP puncta with positive Scarlet and zero Rab2 Intensity:",
num_Scarlet_positive_Rab2_zero, "\n")

# Dataframe Creation
df <- data.frame(
  "HRP puncta" = c("mScalet (-), Rba2(-)", "mScalet (+), Rba2(+)", "mScalet (-), Rba2(+)",
"mScalet (+), Rba2(-)", "mScalet (-), Rba2(-)", "mScalet (+), Rba2(+)", "mScalet (-), Rba2(+)",
"mScalet (+), Rba2(-)"),
  "Genotype" = c("Nrx1AT", "Nrx1AT", "Nrx1AT", "Nrx1AT", "Nrx1", "Nrx1", "Nrx1", "Nrx1"),
  "Value" = c(207, 614, 344, 190, 258, 5, 1079, 1)
)

# Display the dataframe
df

# Define the desired order for the groups (stacks)
group_order <- c("mScalet (-), Rba2(-)", "mScalet (-), Rba2(+)", "mScalet (+), Rba2(+)",
"mScalet (+), Rba2(-)")

# Create the stacked bar chart with the specified order for the groups
ggplot(df, aes(x = Genotype, y = Value, fill = reorder(`HRP puncta`, match(`HRP puncta`,
group_order)))) +
  geom_col() +
  labs(x = "Genotype", y = "Value", fill = "HRP puncta") +
  scale_fill_manual(values = c("mScalet (-), Rba2(-)" = "yellow", "mScalet (-), Rba2(+)" = "cyan",
"mScalet (+), Rba2(-)" = "magenta", "mScalet (+), Rba2(+)" = "blue")) +
  ggtitle("HRP puncta composition")
```
