# Supplementary material for: Versatile nanobody-based approach to image, track and reconstitute functional Neurexin-1 in vivo
Source: Nat Commun. 2024 Jul 18;15:6068. doi: 10.1038/s41467-024-50462-2 (PMC11258300; doi:10.1038/s41467-024-50462-2)
Supplement: Supplementary file 2 — Reporting Summary [file 41467_2024_50462_MOESM2_ESM.pdf]

nature portfolio

Corresponding author(s): Mihaela Serpe

Last updated by author(s): June 27, 2024

Reporting Summary

Nature Portfolio wishes to improve the reproducibility of the work that we publish. This form provides structure for consistency and transparency in reporting. For further information on Nature Portfolio policies, see our Editorial Policies and the Editorial Policy Checklist.

Statistics

For all statistical analyses, confirm that the following items are present in the figure legend, table legend, main text, or Methods section.

n/a	Confirmed	
☐	☒	The exact sample size (n) for each experimental group/condition, given as a discrete number and unit of measurement
☐	☒	A statement on whether measurements were taken from distinct samples or whether the same sample was measured repeatedly
☐	☒	The statistical test(s) used AND whether they are one- or two-sided<br>*Only common tests should be described solely by name; describe more complex techniques in the Methods section.*
☒	☐	A description of all covariates tested
☐	☒	A description of any assumptions or corrections, such as tests of normality and adjustment for multiple comparisons
☐	☒	A full description of the statistical parameters including central tendency (e.g. means) or other basic estimates (e.g. regression coefficient) AND variation (e.g. standard deviation) or associated estimates of uncertainty (e.g. confidence intervals)
☐	☒	For null hypothesis testing, the test statistic (e.g. F, t, r) with confidence intervals, effect sizes, degrees of freedom and P value noted<br>*Give P values as exact values whenever suitable.*
☒	☐	For Bayesian analysis, information on the choice of priors and Markov chain Monte Carlo settings
☒	☐	For hierarchical and complex designs, identification of the appropriate level for tests and full reporting of outcomes
☐	☒	Estimates of effect sizes (e.g. Cohen's d, Pearson's r), indicating how they were calculated

Our web collection on statistics for biologists contains articles on many of the points above.

Software and code

Policy information about availability of computer code

Data collection	Confocal images were acquired using a Zeiss LSM 780 direct confocal microscope (CarlZeiss) and a 40X ApoChromat oil immersion objective, NA=1.4, or a Leica SP8 microscope with a white-light laser and an 100× oil immersion objective, NA=1.4 (HCX PL APO STED white; Leica Microsystems). Live imaging was performed with a Zeiss LSM 880 2-photon confocal microscope using a 40X ApoChromat 1.0 DIC objective. Images were acquired with ZEN Software 2012 (Zeiss LSM 780 and LSM 880) or LAS X Software v. 3.4.2 (Leica SP8). Maximum intensity projections and final composite images were created using ImageJ/Fiji (https://fiji.sc/).<br><br>Electrophysiology recordings were collected using Clampex (pClamp), v 10.7.0.4, Molecular Devices.
Data analysis	MacVector Software, v 18.5, https://www.macvector.com/ RRID:SCR_015700<br>GraphPad Prism 9, v 9.4.1, Graphpad, http://www.graphpad.com/ RRID:SCR_002798<br>ImageJ (Fiji), NIH, v 2.9.0, https://fiji.sc/ RRID:SCR_002285<br>Imaris Software, v 9.7.0, Oxford Instruments https://imaris.oxinst.com/ RRID:SCR_007370<br>LAS X Software v 3.4.2, Leica Microsystems, https://www.leica-microsystems.com/products/microscope- software/details/product/leica-las-x-ls// RRID:SCR_013673<br>Huygens Professional Software, v 15.10.1, https://svi.nl/Huygens-Professional/ RRID:SCR_014237<br>R v 4.2.3, https://www.r-project.org/<br>RStudio v1.4.1717, https://posit.co/products/open-source/rstudio/<br>ggplot2 v 3.5.2, https://ggplot2.tidyverse.org/<br>ggExtra v 0.10.1, https://github.com/daattali/ggExtra<br>RColorBrewer v 1.1-3, https://cran.rstudio.com/web/packages/RColorBrewer/index.html

cowplot v 1.1.3, https://wilkelab.org/cowplot/
ggpubr v 0.6.0, https://rpkgs.datanovia.com/ggpubr/
pClamp (Clampfit), v 11.2.2.17, Molecular Devices, http://www.moleculardevices.com/products/software/pclamp.html/ RRID:SCR_011323
pClamp (Clampex) v 10.7.0.4, Molecular Devices, http://www.moleculardevices.com/products/software/pclamp.html/ RRID:SCR_011323
Mini Analysis, v 6.0.3, Synaptosoft, http://www.synaptosoft.com/MiniAnalysis/ RRID:SCR_002184

For manuscripts utilizing custom algorithms or software that are central to the research but not yet described in published literature, software must be made available to editors and reviewers. We strongly encourage code deposition in a community repository (e.g. GitHub). See the Nature Portfolio guidelines for submitting code & software for further information.

Data

Policy information about availability of data

All manuscripts must include a data availability statement. This statement should provide the following information, where applicable:

- Accession codes, unique identifiers, or web links for publicly available datasets
- A description of any restrictions on data availability
- For clinical datasets or third party data, please ensure that the statement adheres to our policy

Data Availability
Supplementary Figs 1-17 together with Supplementary Table 1, containing average values for the electrophysiological recordings, and Supplementary Table 2, including the experimental conditions for immunohistochemistry, are available in the Supplementary Information file. Any additional information required to reanalyze the data reported in this paper is available from the corresponding author upon request. Source data are provided with this paper.

Code availability
The code facilitating the image data analysis in the current study is available in the Supplementary Information file.

Human research participants

Policy information about studies involving human research participants and Sex and Gender in Research.

Reporting on sex and gender	N/A
Population characteristics	N/A
Recruitment	N/A
Ethics oversight	N/A

Note that full information on the approval of the study protocol must also be provided in the manuscript.

Field-specific reporting

Please select the one below that is the best fit for your research. If you are not sure, read the appropriate sections before making your selection.

[x] Life sciences [ ] Behavioural & social sciences [ ] Ecological, evolutionary & environmental sciences

For a reference copy of the document with all sections, see nature.com/documents/nr-reporting-summary-flat.pdf

Life sciences study design

All studies must disclose on these points even when the disclosure is negative.

Sample size	Sample sizes are in accordance with previous publications and matched with those generally employed in the field.
Data exclusions	All intracellular recordings were performed at room temperature from muscle cells with an initial Vm between –60 and –80 mV and input resistances of ≥ 5 MΩ. mEJP (minature excitatory junction potentials) with a slow rise and falling time, likely arising from neighboring electrically coupled cells, were excluded from analysis.
Replication	Immunohistochemistry experiments and electrophysiological recordings were performed in multiple animals for each genotype. The immunohistochemistry experiments were repeated at least three times over multiple days; the number of data points is indicated in the figure legends and in the Source Data files. Representative images were selected for the figures.
Randomization	No randomization method was used. For immunohistochemistry experiments, all genotypes were processed together, stained in the same tube, mounted on the same slide and imaged in the same session. All experiments were performed at least three times over multiple days. Images were analyzed in an unbiased manner.
Blinding	N/A- Immunohistochemistry experiments, electrophysiological recordings and data analyses were performed in an unbiased manner in

identical conditions. Blinding was not possible for practical reasons as too few investigators were involved in the study.

Reporting for specific materials, systems and methods

We require information from authors about some types of materials, experimental systems and methods used in many studies. Here, indicate whether each material, system or method listed is relevant to your study. If you are not sure if a list item applies to your research, read the appropriate section before selecting a response.

Materials & experimental systems

n/a	Involved in the study
☐	☒ Antibodies
☒	☐ Eukaryotic cell lines
☒	☐ Palaeontology and archaeology
☐	☒ Animals and other organisms
☒	☐ Clinical data
☒	☐ Dual use research of concern

Methods

n/a	Involved in the study
☒	☐ ChIP-seq
☒	☐ Flow cytometry
☒	☐ MRI-based neuroimaging

Antibodies

Antibodies used

Primary antibodies:
Rabbit polyclonal anti-Nrx-1 (C-terminal); Chen et al., 2010
FluoTag-X2 anti-ALFA, NanoTag Biotechnologies, Cat # N1502, RRID:AB_3075985
FluoTag-Q anti-RFP, NanoTag Biotechnologies, Cat # N0401, RRID:AB_2905529
FluoTag-X4 anti-GFP, NanoTag Biotechnologies, Cat # N0304, RRID:AB_2744629
Chicken polyclonal anti-GFP, Abcam, Catalog # ab13970, RRID:AB_300798
Mouse monoclonal anti-Brp (clone nc82), Developmental Studies Hybridoma Bank, Cat # nc82, RRID:AB_2314866
Mouse monoclonal CSP (clone 6D6), Developmental Studies Hybridoma Bank, Cat # DCSP-2 (6D6), RRID:AB_528183
Mouse monoclonal anti-calnexin99A (clone Cnx99A 6-2-1), Developmental Studies Hybridoma Bank, Cat # Cnx99A 6-2-1, RRID:AB_2722011
Goat anti-HRP-FITC, Jackson ImmunoResearch, Cat# 123-095-021, RRID: AB_2314647
Goat anti-HRP-647, Jackson ImmunoResearch, Cat# 123-605-021, RRID:AB_2338967
Goat anti-HRP-405, Jackson ImmunoResearch, Cat# 123-475-021, RRID:AB_2632561

Secondary antibodies:
Goat anti-mouse Alexa-Fluor-488, Thermo Fisher, Cat# A11001, RRID:AB_2534069
Goat anti-mouse Alexa-Fluor-568, Thermo Fisher, Cat# A11004, RRID:AB_2534072
Goat anti-mouse Alexa-Fluor-647, Thermo Fisher, Cat# A21235, RRID:AB_2535804
Goat anti-rabbit Alexa-Fluor-488, Thermo Fisher, Cat# A11008, RRID:AB_143165
Goat anti-rabbit Alexa-Fluor-568, Thermo Fisher, Cat# A11011, RRID:AB_143157
Goat anti-chicken Alexa-Fluor-488, Thermo Fisher, Cat# A11039, RRID:AB_2534096

Validation

The custom anti-Nrx-1 (C-terminal) antibody, a gift received from Janel Richmond (UIC), has been previously validated for use in Drosophila immunohistochemistry (Chen et al, 2010).

All the other antibodies are from commercially available sources and have been validated by the manufacturer with supporting publications found on manufacturer websites, as follows:
FluoTag-X2 anti-ALFA, https://nano-tag.com/product/fluotag-x2-anti-alfa/
FluoTag-Q anti-RFP, https://nano-tag.com/product/fluotag-q-anti-rfp/
FluoTag-X4 anti-GFP, https://nano-tag.com/product/fluotag-x4-anti-gfp/
Chicken polyclonal anti-GFP, https://www.abcam.com/products/primary-antibodies/gfp-antibody-ab13970.html
Mouse monoclonal anti-Brp, https://dshb.biology.uiowa.edu/nc82
anti-calnexin99A, https://dshb.biology.uiowa.edu/Cnx99A-6-2-1
anti-CSP, https://dshb.biology.uiowa.edu/DCSP-2-6D6
Goat anti-HRP-FITC, https://www.jacksonimmuno.com/catalog/products/123-095-021
Goat anti-HRP-647, https://www.jacksonimmuno.com/catalog/products/123-605-021
Goat anti-HRP-405, https://www.jacksonimmuno.com/catalog/products/123-475-021

Goat anti-mouse Alexa-Fluor-488, https://www.thermofisher.com/antibody/product/Goat-anti-Mouse-IgG-H-L-Cross-Adsorbed-Secondary-Antibody-Polyclonal/A-11001
Goat anti-mouse Alexa-Fluor-568, https://www.thermofisher.com/antibody/product/Goat-anti-Mouse-IgG-H-L-Cross-Adsorbed-Secondary-Antibody-Polyclonal/A-11004
Goat anti-mouse Alexa-Fluor-647, https://www.thermofisher.com/antibody/product/Goat-anti-Mouse-IgG-H-L-Cross-Adsorbed-Secondary-Antibody-Polyclonal/A-21235
Goat anti-rabbit Alexa-Fluor-488, https://www.thermofisher.com/antibody/product/Goat-anti-Rabbit-IgG-H-L-Cross-Adsorbed-Secondary-Antibody-Polyclonal/A-11008

	Goat anti-rabbit Alexa-Fluor-568, https://www.thermofisher.com/antibody/product/Goat-anti-Rabbit-IgG-H-L-Cross-Adsorbed-Secondary-Antibody-Polyclonal/A-11011<br>Goat anti-chicken Alexa-Fluor-488, https://www.thermofisher.com/antibody/product/Goat-anti-Chicken-IgY-H-L-Secondary-Antibody-Polyclonal/A-11039

Animals and other research organisms

Policy information about studies involving animals; ARRIVE guidelines recommended for reporting animal research, and Sex and Gender in Research

Laboratory animals	Male or female Drosophila melanogaster larvae were examined in all experiments at the third instar larval stage. The following mutants and transgenic lines were used:<br><br>Drosophila melanogaster: Wild type - w1118<br>Drosophila melanogaster: Nrx[273]/TM6,Tb<br>Drosophila melanogaster: Nrx [Df]/TM6,Tb<br>Drosophila melanogaster: UAS-Nrx-1 (docked at vk37)<br>Drosophila melanogaster: UAS-Nrx-1-AT (docked at vk37)<br>Drosophila melanogaster: UAS-Nrx-1-GFP (Chen et al., 2010)<br>Drosophila melanogaster: Nrx-1-AT<br>Drosophila melanogaster: Nrx-dPDZ-AT<br>Drosophila melanogaster: UAS-Nb-PDZ (random insertion on chromosome II)<br>Drosophila melanogaster: UAS-Nb-mScarlet (random insertion on chromosome III)<br>Drosophila melanogaster: UAS-Nb-mScarlet (random insertion on chromosome II)<br>Drosophila melanogaster: MiMIC [Trojan-Gal4] Nrx/TM6,Tb; RRID: BDSC_67489<br>Drosophila melanogaster: UASp-YFP.Rab2.Q65L; RRID:BDSC_9760<br>Drosophila melanogaster: BG380-Gal4 (X); RRID:BDSC_42736<br>Drosophila melanogaster: ppk-GAL4 (II); RRID:BDSC_32078<br>Drosophila melanogaster: UAS-GFP.nls (II); RRID: BDSC_4775<br>Drosophila melanogaster: UAS-CD4-tdTom (III); RRID: BDSC_35837<br>Drosophila melanogaster: TubP-Gal80[ts] (II); RRID: BDSC_7108<br>Drosophila melanogaster: UAS-Nb-PDZ (docked at VK37)<br>Drosophila melanogaster: UAS-Nb-PDZ (docked at VK37)
Wild animals	This study did not involve wild animals.
Reporting on sex	These analyses did not take in consideration the sex of the Drosophila third instar larvae analyzed.
Field-collected samples	This study did not include samples collected from the field.
Ethics oversight	N/A

Note that full information on the approval of the study protocol must also be provided in the manuscript.
