# Supplementary material for: Versatile nanobody-based approach to image, track and reconstitute functional Neurexin-1 in vivo
Source: Nat Commun. 2024 Jul 18;15:6068. doi: 10.1038/s41467-024-50462-2 (PMC11258300; doi:10.1038/s41467-024-50462-2)
Supplement: Supplementary file 3 — Description of Additional Supplementary Files [file 41467_2024_50462_MOESM3_ESM.pdf]

Supplementary Movie 1.
Bi-directional traffic of mScarlet-labeled vesicles along axons.
Two-photon confocal time-lapses of bundles of motor neuron axons imaged each 5 seconds for a total of 10 minutes. The images collected were converted to movie files at 7 frames/second.
Genotype: <i>UAS-Nrx-1-AT/+; Nrx-1-Gal4/ UAS-Nb-mScarlet</i>

Supplementary Movie 2.
No mobile vesicles are visible in control animals.
Two-photon confocal time-lapses of bundles of motor neuron axons imaged each 5 seconds for a total of 10 minutes. The images collected were converted to movie files at 7 frames/second.
Genotype: <i>UAS-Nrx-1/+; Nrx-1-Gal4/ UAS-Nb-mScarlet</i>

Supplementary Movie 3.
Mobile vesicles in the proximal regions of motor neurons.
Two-photon confocal time-lapses of bundles of motor neuron axons imaged each 5 seconds for a total of 10 minutes. The images collected were converted to movie files at 7 frames/second.
Genotype: <i>BG380-Gal4/Y; tub-Gal80^{ts}/UAS-Nrx-1-AT/+; UAS-Nb-mScarlet/+</i>

Supplementary Movie 4.
Close-up detail from movie 3.
Genotype: <i>BG380-Gal4/Y; tub-Gal80^{ts}/UAS-Nrx-1-AT/+; UAS-Nb-mScarlet/+</i>

Supplementary Movie 5.
Diffuse mScarlet distribution in control animals
Two-photon confocal time-lapses of bundles of motor neuron axons imaged each 5 seconds for a total of 10 minutes. The images collected were converted to movie files at 7 frames/second.
Genotype: <i>BG380-Gal4/Y; tub-Gal80^{ts}/UAS-Nrx-1/+; UAS-Nb-mScarlet/+</i>

Supplementary Movie 6.
Close-up detail from movie 5.
Genotype: <i>BG380-Gal4/Y; tub-Gal80^{ts}/UAS-Nrx-1/+; UAS-Nb-mScarlet/+</i>
